# Efficacy and safety of traditional Chinese medicine retention enema in the treatment of anal sinusitis: A protocol for systematic review and meta-analysis

**DOI:** 10.1097/MD.0000000000032361

**Published:** 2022-12-23

**Authors:** Chunlai Jiang, Jinwu Lu

**Affiliations:** a Department of Anorectal, The Central Hospital of Enshi Tujia and Miao Autonomous Prefecture, Enshi, Hubei Province, China.

**Keywords:** anal sinusitis, meta-analysis, protocol, systematic review, traditional Chinese medicinal enemas

## Abstract

Anal sinusitis is an acute or chronic inflammation of the anal sinus, anal flap, and anal glands. There are many traditional Chinese medicine (TCM) therapies for anal sinusitis, and most of them can achieve satisfactory results. Retention enema is one effective treatment for anal sinusitis. Although a number of studies have shown the effectiveness of retention enema with TCM in treating anal sinusitis, the results are inconsistent. Therefore, a meta-analysis was carried out in this study to evaluate the efficacy and safety of retention enema with TCM in the treatment of anal sinusitis.

**Methods:**

Randomized controlled trials on TCM retention enema for treating anal sinusitis were retrieved from PubMed, EMBASE, The Cochrane Library, CNKI, WanFang databases and VIP databases. The search time limit was from the database establishment to November 15, 2022. Two researchers independently screened the literature, extracted the data, and evaluated the risk of bias in the included studies. Risk of bias was assessed using the Cochrane Risk of Bias Tool (RoB 2.0). The meta-analysis was conducted by RevMan 5.3.

**Results:**

The results of this study will be published in a peer-reviewed publication.

**Conclusion:**

This systematic review and meta-analysis will provide evidence for the efficacy and safety of TCM retention enema in the treatment of anal sinusitis.

## 1. Introduction

Anal sinusitis, also known as anal cryptitis, refers to the acute and chronic inflammatory diseases at the anal sinus and anal flap,^[1]^ with such pathological changes as partial congestion, edema, exudation and tissue hyperplasia.^[2]^ Its clinical manifestations are dull or tingling anal pain, anal distension or foreign body sensation, anal itching and other discomfort.^[3]^ When the sphincter is stimulated, the pain may worsen and affect the buttocks, perineum, sacral tail, posterior femoral and other body parts simultaneously, causing pain and discomfort or difficulty urinating.^[4]^ Due to the special anatomical structure, feces are easily stuck in the anal sinus, leading to bacterial reproduction and inflammation.^[5]^ According to the statistics data, about 85% of patients with anal abscess, anorectal fistula, and anal papillary hypertrophy are caused by anal sinus infection.^[6]^ The early symptoms of anal sinusitis are relatively mild and thus often ignored. Consequently, this disease is apt to recur, refractory and protracted, and eventually develops into chronic inflammatory diseases. Therefore, the early diagnosis and treatment of this disease are of great significance.

Currently, the conventional treatment of anal sinusitis mainly includes Western medicine, traditional Chinese medicine (TCM), acupuncture and surgical treatment.^[7]^ Most of the Western medicine treatment methods control the disease with antibiotics, showing a short-lived effect. As a result, the disease is difficult to heal and easy to relapse, with a prolonged course. Besides, inflammation cannot be cleared.^[8]^ Surgical treatment is so invasive that it is not acceptable to patients.^[9]^ Therefore, it is particularly important to develop a safe and effective treatment.

TCM is a more conservative treatment for anal sinusitis. TCM treatment includes oral, anal plug, fumigation, enema, and acupuncture therapies. Enema therapy targets the disease focus, showing a quick effect.^[10]^ TCM retention enema can act directly on the affected sites and be absorbed by the mucosa, thereby eliminating the first-pass effect and improving bioavailability. Moreover, drug delivery without passing through the gastrointestinal tract reduces adverse drug reactions.^[11]^ Although Western medicine enema has a good therapeutic effect, patients are prone to drug resistance and such adverse reactions as flushing, lower limb swelling, excitement, insomnia, and irritability in the process of treatment.^[10]^ Therefore, its clinical application has been limited. By combining a variety of drugs, TCM enema exhibits a good therapeutic effect, and it causes no adverse reactions in the treatment process. Thus, TCM enema can be widely applied and promoted in clinical practice.

In recent years, an increasing number of studies have been conducted on the treatment of anal sinusitis with TCM retention enema.^[12–15]^ However, there are controversies on its efficacy among different studies, and the evidence is insufficient. Therefore, a meta-analysis was made in this study to evaluate the effectiveness and safety of TCM retention enema in the treatment of anal sinusitis. This paper aims to provide a reference for the clinical treatment of anal sinusitis.

## 2. Methods

### 2.1. Protocol registration

This research protocol was registered in PROSPERO (registration number: CRD42022373380). The protocol report was based on the Preferred Reporting Items for Systematic Reviews and Meta-Analyses Protocols statement guidelines.^[16]^

### 2.2. Inclusion criteria

#### 2.2.1. Types of participants.

The patients who were diagnosed with anal sinusitis according to recognized diagnostic and therapeutic criteria were enrolled. The patient’s gender, age and source were not limited.

#### 2.2.2. Types of interventions.

The experimental group was treated with TCM retention enema. The control group received Western medicine retention enema.

#### 2.2.3. Types of outcome measures.

The primary outcome was clinical efficacy.

Secondary outcome measures were the anal pain score, anal edema relief time, anal sinus congestion and edema score, C-reactive protein, interleukin-8, interleukin-6, tumor necrosis factor-α and adverse reactions.

#### 2.2.4. Types of studies.

Randomized controlled trials published in Chinese or English on the treatment of anal sinusitis with TCM retention enema were included.

### 2.3. Exclusion criteria

1. Repeated studies.
2. Animal experiments.
3. Case reports, retrospective studies and reviews.

### 2.4. Search strategy

Randomized controlled trials on the treatment of anal sinusitis with TCM retention enema were collected from PubMed, EMBASE, The Cochrane Library, CNKI, WanFang databases and VIP databases. The retrieval time was from the database establishment to November 15, 2022. The search strategy of PubMed is shown in Table 1. This strategy is applicable to other electronic databases.

**Table 1:** Search strategy in PubMed database.

Number	Search terms
#1	Anal sinusitis [Title/Abstract]
#2	Anal cryptitis [Title/Abstract]
#3	OR/1-2
#4	Medicine, Chinese Traditional [MeSH]
#5	Chinese Medicine, Traditional [Title/Abstract]
#6	Chung I Hsueh [Title/Abstract]
#7	Traditional Medicine, Chinese [Title/Abstract]
#8	Zhong Yi Xue [Title/Abstract]
#9	Chinese Traditional Medicine [Title/Abstract]
#10	Traditional Chinese Medicine [Title/Abstract]
#11	Hsueh, Chung I [Title/Abstract]
#12	Chinese herbal medicine [Title/Abstract]
#13	Chinese Herbs [Title/Abstract]
#14	Herbal medicine [Title/Abstract]
#15	OR/4-14
#16	Enema [MeSH]
#17	Enemata [Title/Abstract]
#18	Enemas [Title/Abstract]
#19	Enematas [Title/Abstract]
#20	Retention enema [Title/Abstract]
#21	OR/16-20
#22	Randomized Controlled Trial [MeSH]
#23	Controlled trial [Title/Abstract]
#24	Random* [Title/Abstract]
#25	Controlled Clinical Trial [Title/Abstract]
#26	Clinical Trial [Title/Abstract]
#27	OR/22-26
#28	#3 AND #15 AND #21 AND #27

### 2.5. Study selection

**Figure 1.**
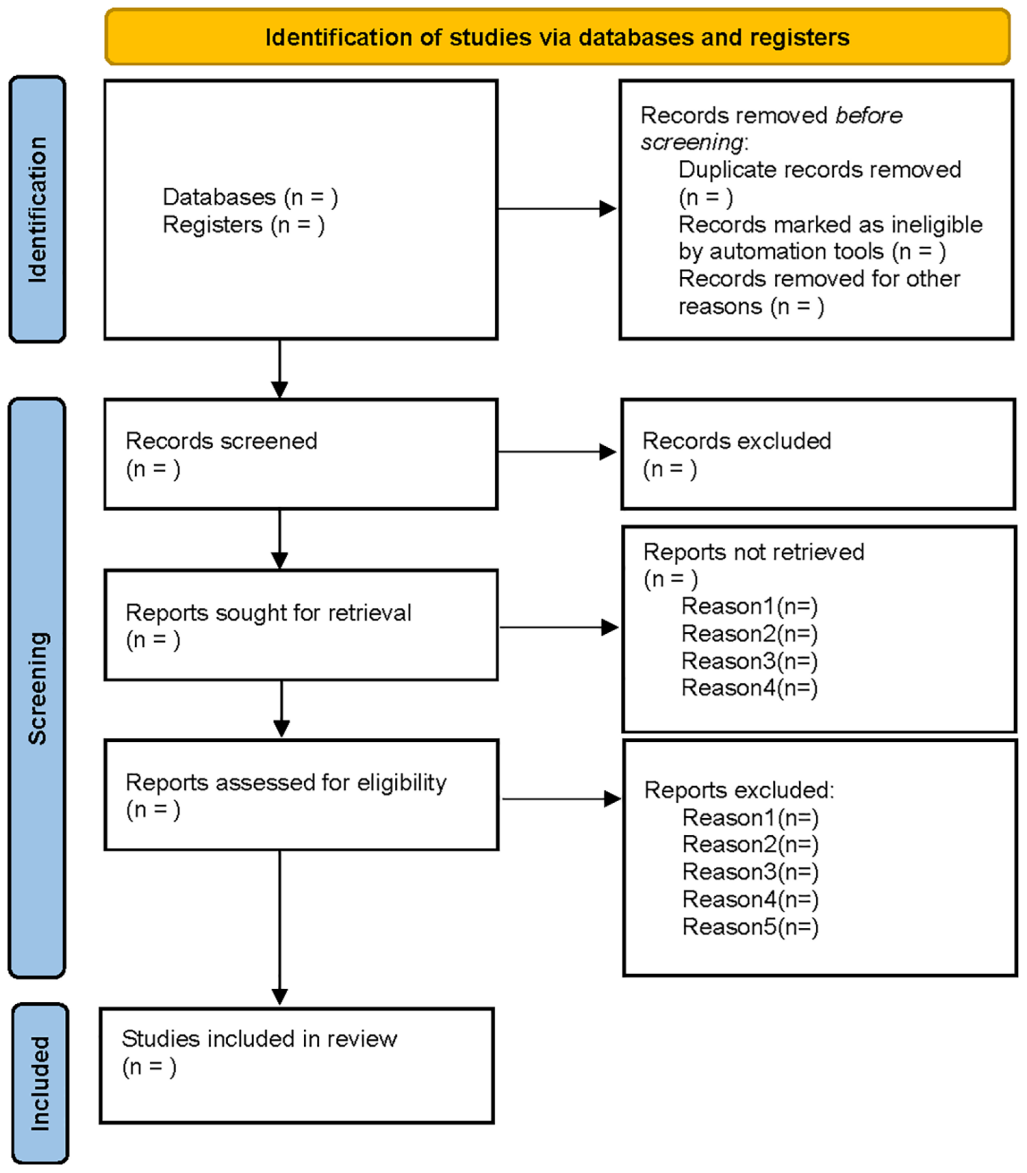
Flow diagram of study selection process.

Two evaluators independently screened the studies, extracted the data and cross-checked them. In case of disagreement, a third party was consulted. During literature screening, after excluding the irrelevant literature, the evaluators read the title and abstract first, and then further examined the full text. Finally, they determined whether to include the study. The screening flow chart of this study was presented in Figure 1.

### 2.6. Data collection and management

The 2 researchers used Excel to extract such information as the paper title, first author, publication time, sample size, age range, treatment duration, therapeutic schedule, outcome indicators, etc. After data extraction, researchers compared the results and discussed differences. A third researcher was consulted to help make a decision if necessary.

### 2.7. Assessment of risk of bias

Risk of bias was assessed using the Cochrane Risk of Bias Tool (RoB 2.0). This tool covers 5 domains: the bias caused by the randomization process, deviations from the established intervention, missing outcome data, outcome measures, and selective reporting of outcomes.

### 2.8. Data synthesis

#### 2.8.1. Data synthesis and heterogeneity assessment.

RevMan 5.3 software was employed for statistical analysis. Dichotomous variables were expressed with the relative risk and 95% confidence interval. Continuous variables were represented by the mean difference and 95% confidence interval. The heterogeneity was analyzed by *χ*^2^ test. If *I*^2^ ≤ 50%, the heterogeneity among included studies was considered small, and the fixed effects model was adopted for analysis. If *I*^2^ > 50%, there was a significant heterogeneity among the included studies, and the random effects model was employed in analysis.

#### 2.8.2. Assessment of reporting bias.

The publication bias was investigated by funnel plots if no less than 10 papers were included.^[17]^

#### 2.8.3. Subgroup analysis.

Subgroup analysis was conducted according to the intervention time and the TCM type.

#### 2.8.4. Sensitivity analysis.

Low-quality literature and studies with large weight were excluded for sensitivity analysis to test the reliability of meta-analysis results.

#### 2.8.5. Grading the quality of evidence.

In order to summarize the evidence and clarify the quality of evidence, the quality of related outcome indicators were rated by the GRADE evidence evaluation system.^[18]^ The limitation, inconsistency, imprecision, indirectness and publication bias of the studies were evaluated.

#### 2.8.6. Dealing with missing data.

To acquire the missing data, the corresponding author was contacted via email. If the data were not available, a comprehensive analysis of existing data would be conducted and the potential impact of missing information would be reviewed.

#### 2.8.7. Ethics and dissemination.

Since this study is systematic review of published literature, ethical approval is not required. The systematic review and meta-analysis will be published in a peer-reviewed journal.

## 3. Discussion

Anal sinusitis belongs to the “poisoning toxin accumulation in the viscera” category of TCM.^[19]^ TCM holds that this disease is caused by dampness and heat. It often occurs in people who have an unclean diet and take in excessive wine, rich, greasy, hot and spicy food. Endogenous dampness and heat produce in these people, and transfer to the anus.^[20–22]^ TCM retention enema enables the drug to be absorbed into the blood through the rectal mucosa of patients, effectively improving the local blood circulation and giving full play to the local or systemic treatment. It has the advantages of simple operation and mild effects. Several studies have proven the effectiveness of TCM retention enema in treating anal sinusitis.^[12–15]^ However, the conclusions of some studies are controversial, and the evidence-based medical proof is lacking. Therefore, a meta-analysis is made to evaluate the efficacy and safety of TCM retention enema in the treatment of anal sinusitis. This systematic review and meta-analysis can provide a reference for clinical treatment of anal sinusitis.

## Author contributions

**Conceptualization:** Jinwu Lu, Chunlai Jiang.

**Data curation:** Jinwu Lu, Chunlai Jiang.

**Formal analysis:** Chunlai Jiang.

**Funding acquisition:** Jinwu Lu.

**Investigation:** Chunlai Jiang.

**Methodology:** Chunlai Jiang.

**Project administration:** Jinwu Lu.

**Resources:** Chunlai Jiang.

**Software:** Jinwu Lu, Chunlai Jiang.

**Supervision:** Jinwu Lu.

**Validation:** Jinwu Lu.

**Visualization:** Jinwu Lu.

**Writing – original draft:** Jinwu Lu, Chunlai Jiang.

**Writing – review & editing:** Jinwu Lu, Chunlai Jiang.
